# Rottlerin as a therapeutic approach in psoriasis: Evidence from in vitro and in vivo studies

**DOI:** 10.1371/journal.pone.0190051

**Published:** 2017-12-22

**Authors:** Min Min, Bing-Xi Yan, Ping Wang, Lilla Landeck, Jia-Qi Chen, Wei Li, Sui-Qing Cai, Min Zheng, Xiao-Yong Man

**Affiliations:** 1 Department of Dermatology, Second Affiliated Hospital, Zhejiang University School of Medicine, Hangzhou, China; 2 Ernst von Bergmann General Hospital, Teaching Hospital of Charité– Humboldt University, Potsdam, Germany; INSERM, FRANCE

## Abstract

Rottlerin is a natural polyphenolic compound that was initially indicated as a PKCδ inhibitor. However, it was recently revealed that it may target a number of molecules and have biological effects on various cell types and is considered as a possible agent for tumor and cell proliferative diseases. Psoriasis is a chronic inflammatory cutaneous disorder with undefined etiology and is characterized by abnormal cellular proliferation, angiogenesis, and inflammation. Therefore, this paper investigates the regulatory effects of rottlerin on normal human epidermal keratinocytes (NHEKs) and imiquimod (IMQ)-induced psoriasiform (IPI) lesions. In vitro results showed that rottlerin inhibited cell proliferation in NHEKs through growth arrest and NFκB inhibition. It may also induce apoptosis in an autophagy-dependent pathway. We found that rottlerin inhibited human microvascular endothelial cells tube formation on matrigel. Rottlerin also decreased the cell senescence of keratinocytes and intracellular ROS generation, which indicated its antioxidant effect. We also showed that rottlerin affects the expression of keratinocyte proliferation biomarkers. In 12-O-tetradecanoylphorbol13-acetate (TPA)–induced keratinocytes, rottlerin significantly inhibited the expression of the induced pro-inflammatory cytokines in keratinocytes. An animal experiment provided the corresponding evidence based on this evidence in vitro, by using IPI model, we found that rottlerin could relieve the psoriasiform of BALB/c mice by inhibiting keratinocyte proliferation, inflammatory cell infiltration, and vascular proliferation. In conclusion, our results suggest that rottlerin may prove useful in the development of therapeutic agents against psoriasis. However, the deep mechanism still requires further study.

## Introduction

Psoriasis is considered as a common inflammatory diseases of the skin that is largely driven by Th17 T-cells[1]. It is characterized with hyper-proliferation and abnormal differentiation of epidermal keratinocytes, increased vascularization of the skin and inflammatory cell infiltration[2, 3]. Although the etiology of psoriasis has not yet been fully elucidated, the abnormal production of several inflammatory mediators from immune cells such as TNF-α, IL-6, IL-17, IL-22 and IL-23 are confirmed to play a key role in psoriasis[1, 2, 4, 5]. In recent years, it has been shown that the disturbed epidermal barrier function plays an important role in psoriasis susceptibility[6]. The differentiation of keratinocytes is demonstrated impaired in the psoriatic skin[7]. Involucrin and loricrin are both proteins play key role in formation of the skin barrier and terminal differentiation of epidermis[8]. It is well known that the expression of involucrin is increased and mislocalized in psoriatic epidermis[9, 10]. However, the expression of loricrin was found to be decreased in lesional and non-lesional skin of psoriasis in comparison with normal skin[11].

Rottlerin (also called Kamala or Mallotoxin) is a polyphenolic compound derived from *Mallotus philipinensis* (Euphorbiaceae)[12]. Rottlerin was originally used as an inhibitor of the PKCδ[12]. However, recent studies indicate that rottlerin may not have a direct effect on PKCδ and PKCδ-independent mechanisms of action have been indicated[13, 14].

Purified rottlerin has recently been found to possess a wide range of therapeutic effects, such as anticancer, antifertility, anti-angiogenic, anti-inflammatory, anti-allergic and anti-oxidation effects[15]. Rottlerin also causes growth arrest in different cell types, including pancreatic cancer stem cells[16], MCF-7 breast cancer cells[17], HT-29 human colon adenocarcinoma cells[18], and human immortalized keratinocytes (HaCaT)[19],which is the mechanism involving inhibition of the transcription factor nuclear factor-kappa B (NFκB) nuclear migration and downregulation of cyclin D1. As a result, many researchers have proposed the possible therapeutic effects by rottlerin to treat psoriasis[15]. Therefore, it is important to clarify the effects of rottlerin on keratinocytes and psoriasis in order to propose a new and acceptable intervention for patients with psoriasis requiring long-term of therapy.

The former data is only based on HaCaT cells. Since HaCaT cells are more readily available and do not require time-consuming isolation from tissues, this cell line has been widely used as an alternative for normal human epidermal keratinocytes (NHEKs) and as in vitro model of psoriasis[20–23]. However, these cells bear some genetic alternations and lack potential inter-donor variability. In addition, since the expression of differentiation markers in HaCaT cells in response to cytokines are different from NHEKs, HaCaT cells may have a limitation when studying related inflammatory skin diseases[23]. In order to identify the effects of rottlerin more precisely, NHEKs were employed to evaluate whether rottlerin induces the same effects observed in cancer and immortalized HaCaT cells. Meanwhile, it is well known that the mouse model of imiquimod-induced psoriasiform inflammation (IPI) may develop a T cell-dependent inflammatory skin disease with several similarities to psoriasis, which serves as a well-established model for investigating of the pathogenesis and therapeutic agents of psoriasis[24]. Therefore, we investigated the effects of rottlerin on the IPI of BALB/c mice, focusing on the alterations induced by rottlerin.

This study demonstrates that rottlerin inhibited the proliferation, cell senescence of NHEKs and tube formation on matrigel made of human umbilical vein endothelial cells (HUVECs). We also showed that inflammatory cytokines induced by 12-O-tetradecanolylphorbol13-acetate (TPA) in keratinocytes were significantly inhibited after rottlerin treatment. These results suggest that rottlerin has multiple anti-psoriatic effects in vitro. Furthermore, we showed that imiquimod-induced psoriasiform inflammation was more attenuated by rottlerin in mice. Taken together, our findings presented provide evidence that rottlerin offers insights into possible therapies for the treatment of psoriasis.

## Materials and methods

### Ethics statement

The Animal Care and Use Committee of medical school of Zhejiang University approved all of animal procedures in this study. Human skin (arm) tissues (2 mm×5 mm) were obtained from 12 healthy adult donors (six males and six females,18–55 years old) after consent was obtained under a protocol approved by the Institutional Review Board at the Zhejiang University. Participants for this study signed written consent forms.

### Isolation and culture of NHEKs

NHEKs were established from skin biopsy samples incubated with dispase, as described earlier [25]. Briefly, detached keratinocytes without contamination were seeded onto flasks at a density of 5000 cells/cm^2^ and maintained in Epilife Medium (Gibco, USA) containing human keratinocyte growth supplement (Gibco, USA) with media refreshed every 48–72 hours. The NHEKs were cultured at 37°C and 5% CO_2_ in a humid atmosphere. Experiments were performed at passage 2–3. Keratinocytes were cultured in proliferation (0.03mM Cacl2 for 72h) or in differentiation conditions (1.2 mM Cacl2 for 48h). Keratinocytes cultured in 1.2mM Cacl2 medium were treated with media containing 0μM, 1μM, 5μM, 10μM rottlerin for the final 24 h (for a total of 72 hours of exposure to Ca2+).

### Cell viability assay

Cell viability was determined using both MTS assay and EdU proliferation assay. NHEKs were seeded at a density of 5×10^4^ cells/well in a 96-well plate. After 24 hours, the media were replaced with fresh media containing 0μM, 1μM, 5μM, 10μM rottlerin. Concentrations of DMSO, which were used to dissolve the rottlerin, were maintained at <0.2% (v/v) among different treatments. In our experiment, a DMSO concentration of 0.2% didn’t affect keratinocyte proliferation, differentiation and death balance in the culture conditions. Cellular proliferation was measured after 24h of rottlerin exposure. When running the assay, reagents from a MTS reagent-based kit (Promega, USA) were added directly into the incubation media and incubated at 37°C for 1h. Absorbance at 490 nm was then measured using a plate reader (BioTek, USA). For the EdU proliferation assay, cells were seeded (n = 1×10^4^ cells per well) in 24-well plates. The cells were then incubated under standard conditions in complete media. Cell proliferation was detected using the incorporation of 5-ethynyl-29-deoxyuridine (EdU) with the EdU Cell Proliferation Assay Kit (Invitrogen, USA) according to the manufacturer’s protocol. The cell nuclei were stained with DAPI (Roche) at a concentration of 5ug/ml for 10 min. The proportion of cells that incorporated EdU was determined using by fluorescence microscopy (Leica, German).

### Cell cycle and apoptosis assays

NHEKs were seeded in six-well plates at 1×10^6^cells/well and were treated with rottlerin at the desired concentrations described above. The cells were harvested and subjected to the following assays after 24 h: For the cell cycle assay, the cells were washed twice with ice cold PBS and fixed in 75% ethanol at -20°C overnight. After fixative removal, the cells were incubated with 50 g/mL propidium iodide/Rnase staining buffer (BD Biosciences PharMingen, San Diego, USA) at 37°C for 15 min. A flow cytometry analysis of DNA content was performed using a FACSCalibur flow cytometer (Becton Dickinson, Franklin Lakes, NJ, USA). For the apoptosis assay, the cells were treated with rottlerin at the desired concentrations described above for 48h, then harvested and washed twice with ice-cold PBS, stained with an Annexin V-PE/7AAD apoptosis kit (BD Biosciences, Franklin Lakes, NJ, USA) according to the manufacturer’s instructions, and analyzed using a flow cytometer. FlowJo software (Version 7.6.1, Treestar, Ashland, OR, USA) was used for subsequent analysis.

### Immunofluorescence staining

Immunofluorescence was performed according to our previously published work [25]. NHEKs were cultured and treated with 5μM rottlerin on sterile glass coverslips placed in a 24-well plate. Cells were washed with PBS and fixed with 4% paraformaldehyde (Sigma Aldrich) for 20 min at room temperature (RT). After washes with PBS, 0.5% Triton X-100 (Sigma Aldrich) (v ⁄ v in PBS) was added for 5 min at 4°C and then washed with PBS. Cells were incubated with blocking buffer (2.5% BSA in PBS) for 1h prior to incubation at 4°C overnight with primary antibody. The following antibodies were used for immunofluorescence assay: anti-NFκB p65(1:200, Santa Cruz, SC-372); anti-p53(1:200, Santa Cruz, SC-98); anti-integrinβ1 (1:200, Cell Signaling Technology, 4706s). Secondary antibodies labeled with FITC(Green)/Cy3(Red) (1:5000, Jackson Immuno Research) were used. Cell nuclei were stained with DAPI for 10 minutes at a concentration of 5ug/ml (applied after incubation with secondary antibody). Images were acquired on a an fluorescence microscope (Leica, German). Negative controls stained with secondary antibody alone showed no immunolabelling. A control with no treatment was systematically included.

### HUVECs tube formation assay for in vitro angiogenesis

The human umbilical vein endothelial cells (HUVECs) was purchased from ATCC (Manassas, VA, USA). The 96-well plate were coated with cold matrigel (BD Biosciences, USA). After incubation of 1 h at 37°C, HUVECs (2×10^4^ cells/well) were seeded to matrigel-coated wells and were treated with rottlerin at desired concentrations (1μM, 5μM, 10μM). Four hours later, three non-overlapping microscopic images in each well were randomly photographed at low-power magnification. The observed total tube length and branching points formed by endothelial cells per image field were measured by using Image-Pro Plus software (Media Cybernetics, USA).

### Senescence associated β-galactosidase staining

Before the staining procedure, 1×10^4^ NHEKs were plated in a 24-well plate and treated with rottlerin as above for 24 h. The detection of cellular senescence was performed by using the Senescence Detection Kit (GENMED SCIENTIFICS INC., USA) according to the manufacturer's instructions. The cells were briefly washed with PBS at least three times and then fixed using a fixation buffer at room temperature for 20 min. The cells were then rinsed and washed cells by PBS at least three times, stained by β-galactosidase staining solution, and incubated at 37°C for 10h in a dry incubator. The blue granules observed within the cytoplasm under a light microscopic examination were considered positive for the beta-galactosidase staining, suggesting senescence of the observed cells.

### ROS quantification by Fluorescence Activating Cell Sorter (FACS) analysis

The detection of cellular levels of reactive oxygen species (ROS) was performed by using the ROS Detection Kit (GENMED SCIENTIFICS INC., USA) according to the manufacturer's instructions. NHEKs were plated at a density of 5×10^4^ cells/ml onto six-well plates. Twenty-four hours later, the cells were pretreated with 5 μM rottlerin for 24 h before 100 μM H_2_O_2_ application for 30 min then cells were harvested by trypsinization. The keratinocytes were loaded with 2 μmol·L^−1^ DCFH-DA and incubated at 37°C for 30 min. The fluorescence intensity was monitored using a BD FACSCalibur flow cytometer and the data were analyzed using the FlowJo software.

### Transmission electron microscopy

We further investigated the ultra-structural alterations of NHEKs treated with rottlerin using Transmission electron microscopy (TEM). NHEKs treated with 5μM rottlerin was analyzed using TEM by comparison with control cells. Generally, the cells were harvested and washed with PBS and then fixed in ice-cold 2.5% glutaraldehyde overnight. The cell pellets were dissected and cut into 1–2 mm2 pieces. After being washed with PBS three times for 15 min, the cells were post-fixed in 1% OsO_4_ for 1 h and stained with 2% uranyl acetate for 30 min at room temperature. Then cells were then dehydrated using a graded series of ethanol (50, 70 and 90%) for 15 min each, ethanol (100%) for 20 min, and 100% acetone for 20 min, respectively. Sections of 70-nm thickness were placed on copper grids (Leica) and imaged using a JEM 1200EX transmission electron microscope (JEOL, Tokyo, Japan)

### Western blotting

The Western blot analysis was performed as we described earlier[25].Primary antibodies used were anti-Bax (1:500, Santa Cruz, SC-20067), anti-Bcl2 (1:1000,Santa Cruz, SC-509),anti-cleaved Caspase-3(1:500, Proteintech, 25546-1-AP),anti-LC3A/B(1:500,Cell Signaling Technology Technology, #12741),anti-Beclin-1(1:1000,Cell Signaling Technology, #3495),anti-Atg5(1:1000,Cell Signaling Technology, #12994), anti-Atg12(1:1000,Cell Signaling Technology, #4180), anti-IKKα(1:500, Santa Cruz, SC-11930),anti-IKKβ(1:500, Santa Cruz, SC-8943), anti-NF-κB p65(1:500, Santa Cruz, SC-372),anti-GAPDH(1:2000,Proteintech, 60004-1-lg). Secondary antibodies used in immunoblotting studies were HRP-conjugated (1:5000,Jackson Immuno Research).Signals were revealed by enhanced chemiluminescence kit (Millipore).

### Quantitative real-time PCR

Total RNA was extracted from normal (*n* = 3) keratinocytes. Loricrin, involucrin, TNF-α, IL-6, IL-23 and β-actin mRNA were analyzed by quantitative real-time PCR (qRT-PCR). β-actin was used as an internal control, and its expression was not altered by rottlerin. The primers used for PCR were designed by Beacon Designer v. 8.0 (Premier Software) and are listed in the electronic supplementary material, S1 Table. The quality and quantity of total RNA were assayed using a NanoDrop instrument (NanoDrop Technologies, Wilmington, DE). A total of 2 μg RNA was reversed into cDNA by using Superscript II Reverse Transcriptase (Invitrogen). The qRT-PCR was performed using an ABI StepOne Plus Fast Real-Time PCR system (Applied Biosystems, Grand Island, NY) according to the manufacturer’s instructions with the recommended parameters. Gene expression was determined using SYBR green PCR mix (Roche) and 10 ng of template. The miRNAs were reverse transcribed using MicroRNA First Strand Synthesis Kit (TaKaRa). Then, SYBR Premix Ex Taq Kit (TaKaRa) and miRNA-specific primers were used for cDNA amplification according to the manufacturer's protocol. U6 was used as an internal control. Negative controls containing water instead of cDNA were performed to ensure purity of all reagents. CT values were analyzed using qBase Plus 2 software (Biogazelle, Zwijnaarde, Belgium). The results were expressed as the fold difference in gene expression relative to the endogenous gene and compared with control samples.

### Enzyme-linked immunosorbent assay (ELISA)

Near-confluent human keratinocytes were treated with 1uM for 24 hours. The cells were co-treated with or without 100 nM TPA for the final 2 hours (for a total of 24 hours of exposure to rottlerin). Cell culture supernatants were collected and stored at -80°C and subjected to a single freeze-thaw cycle. Human TNF-α (no. EK0525), human IL-17(no. EK0430), and human IL-22 (no. EK0933) kits from BOSTER (Wu Han, China) were used. The ELISA assay was performed according to the manufacturer’s instructions.

### Mice

Five-week-old female BALB/c mice with an average bodyweight of approximately 20 g were bought from the Shanghai Laboratory Animal Co. Ltd (Shanghai, China). The mice were fed with normal forage and provided with water ad libitum in the animal experimental center of the Second Affiliated Hospital of Zhejiang University Medical School.

### Mouse model of IPI

The mice were numbered and randomly assigned to two groups in which the corresponding forages were given for 1 week. Then, an area of 4 cm×10 cm was shaved from the back of all the mice. The mice were then given a daily topical dose of 12.5 μg of imiquimod cream (5%) (MED.SHINE, China) for six consecutive days. Rottlerin (20mg/kg) or a vehicle in 100 ul saline was administered to IPI BALB/c mice through gavage administration (once daily) before the topical use of imiquimod. The administration dose of rottlerin was mainly referred to the previous work [26, 27]. Then, the pre-experiment was introduced to determine the optimum dose. Erythema, scale formation, and thickness were analyzed and evaluated separately on a 0–4 severity scale. The three values were then added together to give a total severity score for each mouse (maximum score). The mouse was sacrificed by dislocation of the neck. Samples of skin, spleen, and draining lymph nodes were collected on the sixth day 6 for future experiments.

### Histopathology analyses

Skin samples from BALB/c mice were either fixed in 10% neutral buffered formalin. Formalin fixed skin were processed and embedded in paraffin, sectioned at 5μm, and stained with hematoxylin-eosin. The slides were observed under digital slice scanner (NanoZoomer Digital Pathology Japan). The H&E slides were scored by a semiquantitative analysis for the following parameters: epidermal thickness and angiogenesis.

### Cell preparation

The single-cell suspension of mouse skin preparation was performed as described earlier[28]. Spleens and draining lymph nodes were minced in RPMI/10%FBS (Gibco, USA) through a 200 mesh screen and centrifuged at 800 g for 5 min. Next, red blood cells (RBC) were lysed with 0.1 N NH_4_Cl, and both splenocytes and draining lymph node cells were washed three times and suspended in RPMI/10%FBS.

### Flow cytometry analysis

For each staining, the cells (1×10^6^cells/ml) were fluorescently labeled after incubation in a dark room for 30 min at room temperature with the following antibodies (all from eBioscience, USA): rat-anti-mouse CD3 APC (no. 17-0032-82), rat-anti-mouse CD3 FITC (no. 11-0032-82),rat-anti-mouse CD4 APC (no. 17-0041-82), and rat-anti-mouse CD8aFITC (no.11-0081-82). All of the samples were detected on a BD FACSCalibar flow cytometer and analyzed with FlowJo software.

### Immunohistochemical analyses

Mouse back skin was fixed in 10% formalin and embedded in paraffin. An Immunohistochemical analysis was performed using the standard ABC-peroxidase Kit (Vector, Burlingame, Ontario, CA, USA) as suggested by the manufacturer. Affinity-purified biotinylated anti-rabbit and anti-mouse IgG was purchased from Vector Lab (Burlingame, Ontario, CA, USA). Other antibodies used in this study included CD3 (Abcam, ab16669), CD11b (Abcam,ab133357), CD11c (Abcam,ab11029). A representative picture showing similar results for each group was chosen for publication. Negative controls without primary antibodies showed no immune-labelling.

### Statistical methods

Prism 5 software (GraphPad, SanDiego, CA, USA) was used to perform the statistical analysis. Student’s t-test and one-way ANOVA test were used depending on the experimental conditions. All the data are presented as mean ±SEM (Standard Error of Mean), and P<0.05 was considered statistically significant.

## Results

### Rottlerin inhibits cell proliferation and cell viability in NHEKs.

**Fig 1:**
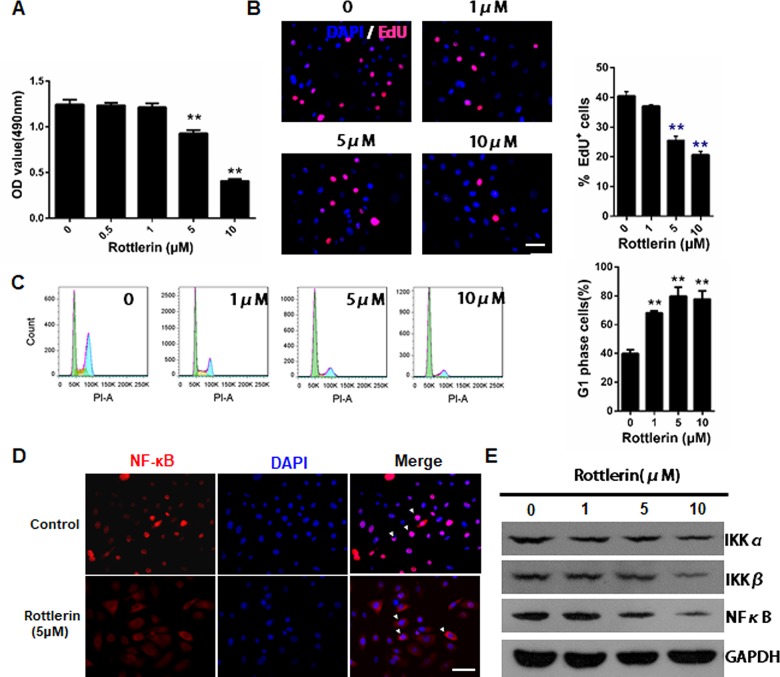
Rottlerin inhibits cell proliferation and cell viability through cell cycle arrest and inhibition of NFκB activation. NHEKs were treated with various concentrations of rottlerin for 24 h. (A) Cell viability was evaluated using a MTS assay. The data are presented as the mean±SEM (n = 5). The data are representative of three independent experiments. **P<0.01 vs. control. (B) Cell proliferation ability was evaluated using EdU staining. The percentage viability (live cell count/total cell count) was calculated and expressed as mean±SEM in five representative fields for each group. **P<0.01 vs. control. The experiment shown is representative of three experiments. (C) A Flow cytometry analysis was used to determine the cell progression and cell cycle distribution. The data are presented as mean±SEM of triplicate experiments. **P<0.01 vs. control. (D) NHEKs were treated with 5μM rottlerin for 12h, then stained with an anti-p65 NFκB subunit antibody and revealed with cy3-conjugated secondary antibody and DAPI (blue). Scale Bar = 20 μm. The images were selected from three individual experiments. (E) A western blotting analysis of total protein extracts showing IKKα, IKKβ and NFκB p65 protein expression in NHEKs treated with 1, 5, and 10μM rottlerin for 24h. GAPDH was used as a loading control. The data are representative of three independent experiments.

The effects of rottlerin on the metabolic activity of NHEKs were evaluated using MTS assay. NHEKs were treated with various concentrations of rottlerin (0–10μM) for 24 h. As shown in Fig 1A, the cell density of rottlerin-treated NHEKs was markedly suppressed and became statistically significant starting from 5μM rottlerin with a reduction of 25% in the control cells (*P*<0.01; Fig 1A). The actual cell proliferation of the rottlerin-treated keratinocytes was then determined by EdU assay. Compared with the control group, the number of EdU-positive cells significantly decreased after rottlerin treatment in a dose-dependent manner (*P*<0.01; Fig 1B), suggesting that rottlerin inhibited the DNA synthesis.

### Rottlerin induces cell cycle arrest and inhibited NFκB activation in NHEKs

A cell cycle analysis was performed via an FACS analysis in order to understand the underlying mechanism of rottlerin-induced cell growth inhibition. Flow cytometry analyses of NHEKs treated with rottlerin (0–10μM) suggested a significantly increase in the cell population during the G0/G1 phase with a concomitant decrease during the G2 and S phases, accompanied by increasing concentrations of rottlerin in NHEKs (*P*<0.01; Fig 1C). Since NFκB plays a key role in cell proliferation and cell cycle, we further investigated whether rottlerin regulates NFκB activation in keratinocytes. Fig 1D shows that the fluorescence of the anti-p65 NFκB was mainly distributed in the nucleus, whereas after 12h of 5μM rottlerin treatment, the fluorescence was mainly confined to the cytoplasm, which indicated rottlerin induced NFκB nucleus translocation inhibition in NHEKs. In addition, as shown in Fig 1E, we found that rottlerin dramatically supressed the expression of total IKKα, IKKβ and NFκB p65 in whole cell extracts, which may be indicative of the nucleus translocation inhibition of NFκB p65 during rottlerin treatment.

### Rottlerin induces apoptosis in NHEKs

**Fig 2:**
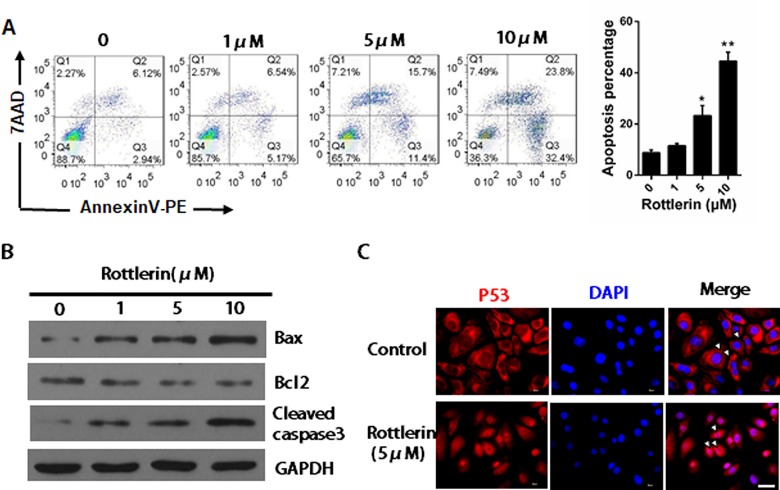
Rottlerin stimulates apoptosis in NHEKs. (A) NHEKs were treated with various concentrations of rottlerin for 48 h. Apoptotic cell-death was measured by annexin V-PE/7AAD staining in NHEKs. The graph represents the mean±SEM of the percent of apoptotic cells in the flow cytometry results. The data are presented as the mean±SEM (n = 3). *P<0.05, **P<0.01 vs. control. The data are representative of three independent experiments. (B) Immunofluorescence analysis of p53 in NHEKs after treatment with 5μM rottlerin for 24h. The cells were stained with an anti-p53 antibody, and revealed with cy3-conjugated secondary antibody and DAPI (blue). The arrows point out that p53 translocate from the cytoplasm to the nucleus. Scale Bar = 20 μm. The images are representative of three independent experiments. (C) Western blotting analysis of Bax, Bcl2, cleaved Caspase-3 protein expression after NHEKs treated with 1, 5, and 10μM rottlerin for 48h. GAPDH was used as the loading control. The data are representative of three independent experiments.

The effect of rottlerin on apoptosis was evaluated using flow cytometry with annexinV-PE/7AAD double staining in order to assess whether rottlerin-induced cell growth inhibition was associated with cell apoptosis. Rottlerin did not significantly induce apoptosis in NHEKs at 24 h (data not known), but it significantly induced apoptosis at 48 h. The percentage of apoptotic NHEKs following a 48 h treatment with 5 or 10 μM rottlerin was 27.1% and 56.2%, respectively, compared with the control group (*P*< 0.05 and *P*<0.01, respectively; Fig 2A). These results correspond to the addition of values that are shown in the higher and lower right quadrants of each panel, which indicate the early and late stages of apoptosis, respectively. The expression levels of apoptosis regulatory proteins were examined in NHEKs using western blot analysis following a 48h treatment with 1, 5 or 10 μM rottlerin. As shown in Fig 2B, the Bax and cleaved Caspased-3 expression levels increased following treatment with rottlerin, compared with the control group, whereas the Bcl-2 expression level decreased in a dose-dependent manner. The translocation of p53 is an important characteristic of apoptosis. The merge images showed that anti-p53 labeling (red) was mainly restricted to the cytoplasm but after 24 h of rottlerin treatment, the p53 expression was mainly restricted in the nucleus (Fig 2C), which indicated that rottlerin affected the subcellular distribution of p53.

### Rottlerin induces autophagy in NHEKs

**Fig 3:**
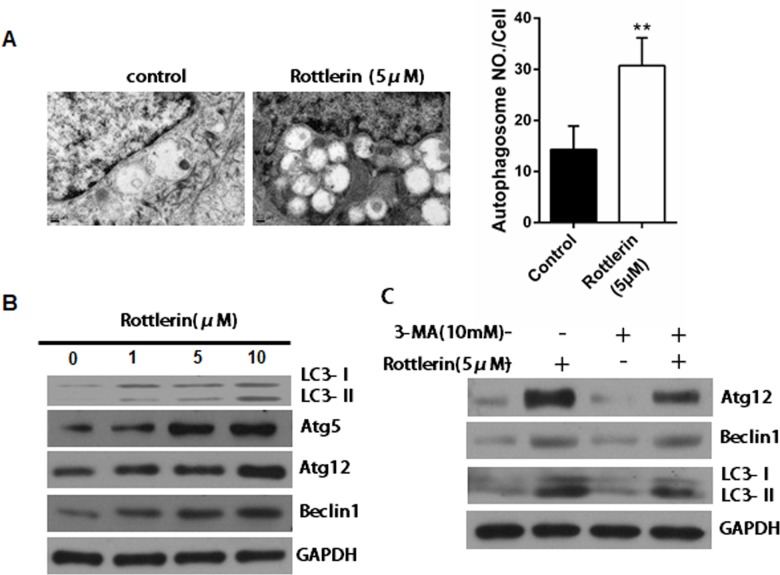
Rottlerin induces autophagy in NHEKs. (A) Transmission electron microscopy (TEM) images showed the ultra-structural features of untreated NHEKs or NHEKs treated with 5μM rottlerin for 24 h. The results are expressed as the average number of autophagosomes structures per cell obtained by examining at least 20 cells. Bar = 0.25 μm. **P<0.01 vs. control. (B) A western blot analysis was performed to measure the expression of LC3, Atg5, Atg12 and Beclin-1 after NHEKs treated with 1, 5 and 10μM rottlerin for 24 h. GAPDH was used as loading control. The data are representative of three independent experiments. (C) NHEKs were pre-incubated with 10mM 3MA for 24 h, followed by treatment with 5μM rottlerin for 24 h. The western blot analysis was performed to measure the expression of Atg12, Beclin-1 and LC3. GAPDH was used as the loading control and the data are representative of three independent experiments.

NHEKs were treated with 5μM rottlerin for 24 h and the ultrastructure of the cells was analyzed by TEM in order to evaluate whether the autophagy was responsible for rottlerin-induced apoptosis. As shown in Fig 3A, numerous autophagic vacuoles containing lamellar structures, or residual digested material and empty vacuoles were observed in the NHEKs when treated with rottlerin. Compared with the control group, the autophagosome number increased about 2.5 fold in NHEKs after rottlerin treatment (*P*<0.01; Fig 3A). Furthermore, the western blot results showed that the expression levels of the autophagy-associated proteins Atg5, Atg12, Beclin1 and the conversion of LC3-I–LC3-II were increased in a dose-dependent manner (Fig 3B). To further confirm that rottlerin induced autophagy in keratinocytes. We tested whether pharmacological inhibition of autophagy by autophagy inhibitor 3-methyladenine (3MA) has impacts on the autophagic process of keratinocytes treated with rottlerin. We exposed the NHEKs to 3MA before treating it with rottlerin in order to confirm the role of rottlerin in cell autophagy. Fig 3C shows the co-treated NHEKs with rottlerin (5μM) and 3-MA (10 mM) inhibited autophagy, as assessed by Atg12, Beclin 1and LC3 through a western blot analysis. These findings suggest that rottlerin treatment can trigger the autophagic process in keratinocytes.

### Rottlerin regulates the expression of proliferation and differentiation-related molecules in NHEKs

**Fig 4:**
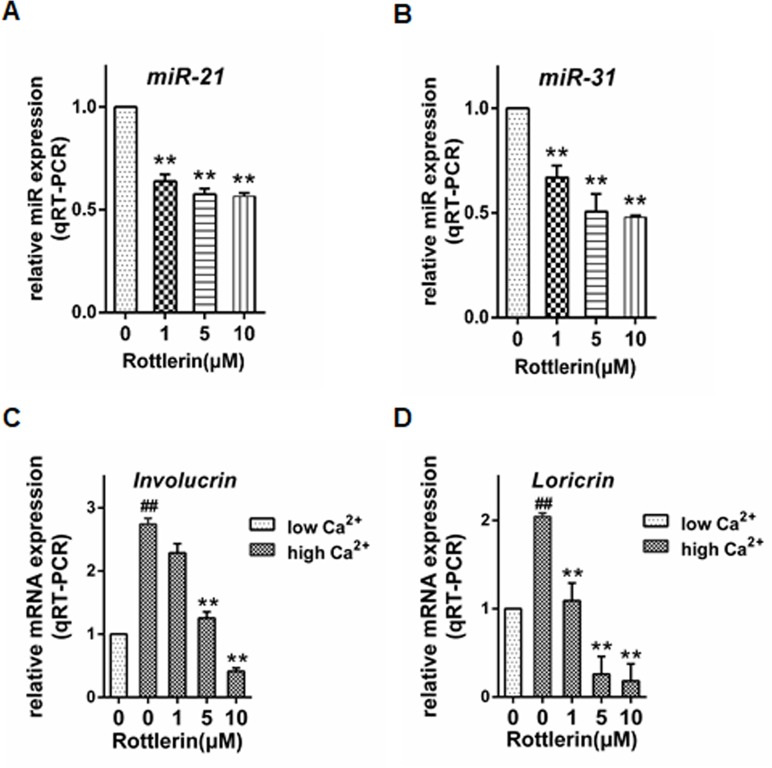
Rottlerin regulates expression of proliferation and differentiation-related molecules in NHEKs. NHEKs were treated with 0, 1, 5, and 10μM rottlerin for 24 hours. The effect of rottlerin on miRNA expression levels of keratinocytes proliferation associated markers were investigated using qRT-PCR. U6 was used as the internal control. (A) miR-21, (B) miR-31 (n = 3, ** *P*<0.01). NHEKs were maintained in proliferation (0.03mM Ca^2+^ for 72h) or in differentiation conditions (1.2mM Ca^2+^ for 48h). Keratinocytes cultured in 1.2mM Ca^2+^ medium were then treated with 0, 1, 5, and 10μM rottlerin for 24h. The effect of rottlerin on mRNA expression levels of keratinocytes differentiation associated markers were investigated using qRT-PCR. Actin was used as the internal control. (C) Involucrin, (D) Loricrin (n = 3, ## p<0.01 versus low Ca^2+^, ** p<0.01 versus high Ca^2+^).

Psoriasis is characterized by abnormal keratinocyte proliferation and differentiation. To further investigate the biological role of rottlerin in psoriasis, the effect of rottlerin on proliferation markers of keratinocytes was examined. Given that miR-21 and miR-31 are both key regulators in keratinocytes proliferation of psoriasis. A qRT-PCR analysis showed that the miRNA expression of miR-21 and miR-31 were significantly down-regulated by rottlerin treatment in a dose-dependent manner (*P*<0.01; Fig 4A and 4B). Then, we examined the effect of rottlerin on the expression of keratinocyte differentiation markers with Ca^2+^ switch assay. When rottlerin was added to the cultures at a concentration of 10μM, the mRNA levels of involucrin were reduced by about 80% (*P*<0.01; Fig 4C). However, our data indicated that rottlerin induced a significant decrease in mRNA expression of loricrin in human keratinocytes with a maximum down-regulation at a concentration of 10μM (*P*<0.05; Fig 4D). These data indicated that rottlerin might inhibit keratinocytes proliferation. However, rottlerin didn’t show the advantage in keratinocytes differentiation restoring.

### Rottlerin exhibits anti-inflammatory effect in TPA-triggered keratinocytes

**Fig 5:**
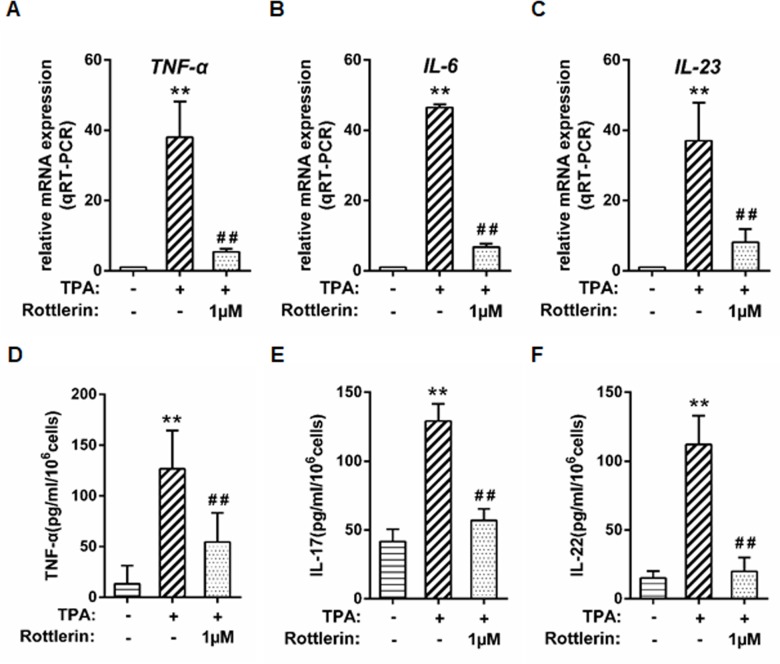
Rottlerin exhibits anti-inflammatory effect in TPA-triggered keratinocytes. Near-confluent cultures of primary keratinocytes were treated with 0, 1, 5, and 10μM rottlerin for 22 hours. The cells were then co-treated with 100nM TPA for 2 hours (for a total of 24 hours of exposure to rottlerin). The mRNA expression levels were measured at 24h by qRT-PCR: (A) TNF-α; (B) IL-6; (C) IL-23. Mediators in the supernatant fluids at 24 h were measured by ELISA: (D) TNF-α; (E) IL-17; (F) IL-22. (n = 3, ** P<0.01 versus control, ^##^ *P*<0.01 versus TPA alone).

Accordingly, we examine the effects of rottlerin on inflammatory action. We opted to examine the effect of rottlerin on the expression of keratinocytes treated with TPA, which is known to activate keratinocytes and induce a release of inflammatory mediators. Near confluent NHEKs were treated with different concentrations of rottlerin for 24 hours. At 22 hours, the cells were spiked with 100 nM TPA (for 2 hours), and mRNA was isolated. The effect of rottlerin on mRNA expression of TNF-α, IL-6 and IL-23 were examined. The qRT-PCR results showed that, in NHEKs, TPA stimulation triggered almost 38-fold increase in the level of TNF-α mRNA, 46-fold increase in the level of IL-6 mRNA level, and 37-fold increase in the level of IL-23 mRNA level as compared to control cells (all *P*<0.01; Fig 5A, 5B and 5C). Rottlerin pretreatment significantly inhibited the TPA-induced expression of TNF-α, IL-6 and IL-23 at a concentration of 1μM, thus revealing the specific anti-inflammatory effect of this compound on keratinocytes (all *P*<0.01; Fig 5A, 5B and 5C). We also confirmed the qRT-PCR results using an ELISA assay to monitor the secretion of mediator release from NHEKs. Near-confluent primary keratinocytes were treated as above and the supernatants were collected for ELISA assay. Similarly, TPA stimulation induced about 9-fold increase in TNF-α release, 3-fold increase in IL-17 release, and 7-fold increase in IL-22 release compared to the control cells (all *P*<0.01; Fig 5D, 5E and 5F). Rottlerin at a concentration of 1μM significantly blocked the release of three inflammatory markers (all *P*<0.01; Fig 5D, 5E and 5F). In conclusion, our data demonstrated that rottlerin displays anti-inflammatory effect on keratinocytes treated with TPA.

### Rottlerin inhibits tube formation in HUVECs for angiogenesis

**Fig 6:**
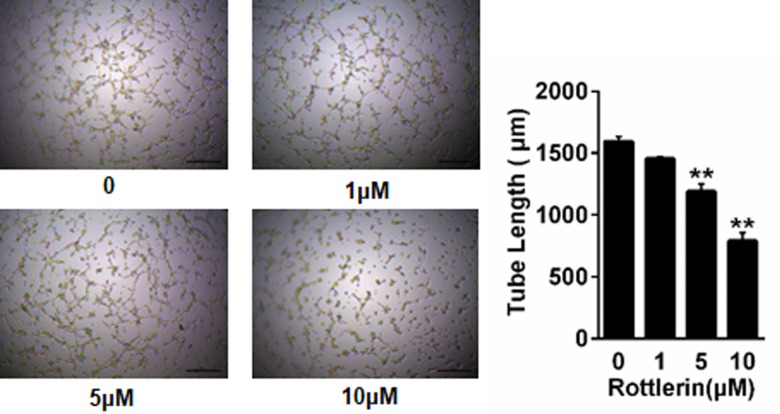
Rottlerin inhibits angiogenesis in HUVECs. Angiogenesis ability was measured using a HUVECs tube formation test. The total length of the tube per field was quantified by counting three random fields or well under the microscope(x4). The data are represented as mean±SEM. ** *P*<0.01 vs. control. The experiment shown is representative of three independent experiments.

Since angiogenesis plays a major role in psoriasis, a tube formation assay was carried out to investigate the influence of rottlerin on endothelial cell differentiation into a capillary-like structure, which is a major step in the angiogenic process. As shown in Fig 6, the untreated HUVECs on matrigel formed an extensive network of thin interconnected tubes, while in rottlerin-treated (5 and 10μM) cells, the tubes appear to be incomplete in a dose-dependent manner (both *P* < 0.01).

### Rottlerin decreases ROS generation in NHEKs

**Fig 7:**
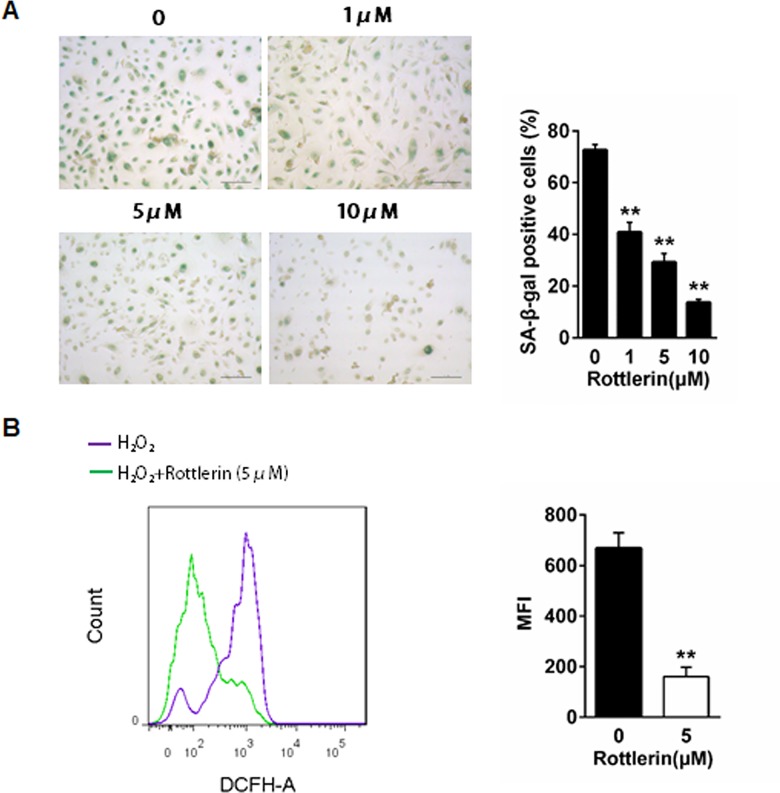
Rottlerin inhibited cell senescence and ROS in NHEKs. (A) NHEKs were pretreated with different concentrations of rottlerin for 24 h. After treatment, the cells were processed for SA-βgal staining. Senescent cells are shown in blue color. Histogram represents the average percentage values of SA β-Gal positive cells in five representative HPFs. Bar = 20μm. ** *P*<0.01 vs. control. Data are representative of three independent experiments. (B) NHEKs were treated with or without rottlerin (5μM) for 24h prior to treatment of H_2_O_2_ (100 μmol·L−1). After 30min, cells were subjected to DCFH-DA FACS. Quantitative intensity of fluorescence in the peak are shown in the histograms. Data are means ± SEM (n = 3 for each group). ** *P*<0.01 vs. control. Similar results are shown in two other independent experiments.

The effects of rottlerin on the anti-oxidant were examined using a SA-β-gal staining assay. The percentage of NHEKs with blue positive staining decreased following treatment with rottlerin for 24 h compared with the untreated cells, and this trend occurred in a dose-dependent manner (*P*<0.01, Fig 7A). Since cell senescence is considered associated with intracellular ROS generation, the present study further examined whether rottlerin could inhibit ROS generation in NHEKs. As shown in Fig 7B, incubation with H_2_O_2_ for 30 min shifted the histogram to the right, which indicated an increase in the level of ROS. However, treatment with 5μM rottlerin for 24 h before H_2_O_2_ application suppressed the increase in the level of ROS induced by H_2_O_2_. Pretreatment of rottlerin significantly reduced the cellular median fluorescent intensities of DCFH-DA (representing intracellular ROS levels) in NHEKs (*P*<0.01; Fig 7B), which indicated that the reduction of DCFH-DA fluorescence by H_2_O_2_ was partly reversed in the presence of rottlerin. Above all, this data showed that rottlerin reduced senescence and ROS accumulation and in NHEKs.

### Rottlerin treatment reduces psoriasiform lesions in IMQ-induced mice

**Fig 8:**
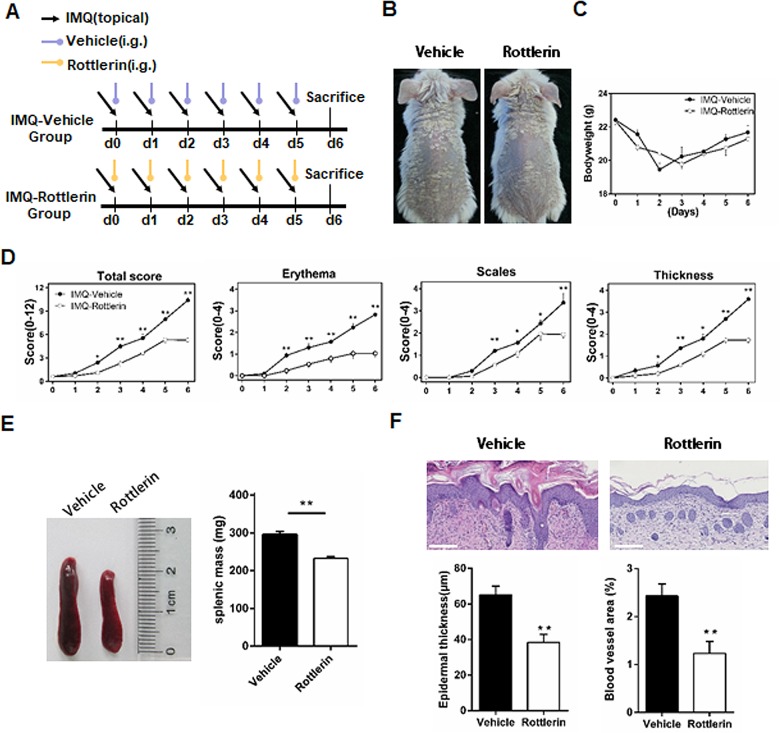
Rottlerin administration attenuates IMQ-treated mice. (A)Protocol for IPI model and dosing of rottlerin. (B) Representative clinical presentations of IMQ-treated mice from the vehicle group (left) and rottlerin group (right). (C) Quantification of body weight changes between the vehicle group and rottlerin group. (D) Scoring was performed daily using the erythema, scales, and thickness elements of the PASI to assign a score of 0–4 to each animal and thereby assess the effects of rottlerin in the IPI mouse model. The data points are presented as the mean±SEM of five mice per group. *P< 0.05, **P< 0.01 vs. vehicle. Data are representative of three independent experiments. (E) The relative size of spleens in the rottlerin group (right) compared with vehicle group (left). The quantification of spleen mass in the two groups is shown as the mean±SEM (n = 5 for each group). **P<0.01 vs. vehicle. The data are representative of three independent experiments. (F) Representative H&E images of dorsal neck skin from IMQ-induced mice treated with vehicle or rottlerin. The average epidermal thickness and blood vessel area of the dorsal neck skin from the vehicle and rottlerin groups are shown. The data are represented as mean±SEM (n = 6 for each group). Scale bar = 100μm. **P<0.01 vs. vehicle. The experiment shown is representative of three independent experiments.

To assess whether rottlerin treatment improved IMQ-induced skin inflammation in vivo, we applied IMQ cream topically to the shaved back skin for six days consecutively with a daily rottlerin (20mg/kg) or vehicle forage (Fig 8A). Mice dosed with rottlerin attenuated IMQ-induced psoriasiform inflammation obviously but did not clear them completely, as compared with the vehicle group (Fig 8B). Body weight changes showed no significant differences between IPI mice fed with a vehicle or rottlerin forage daily (*P*>0.05; Fig 8C). As shown in Fig 6D, the average adjusted PASI (Psoriasis Area and Severity Index) score in the rottlerin-treated group of mice was much lower than that in vehicle-treated group of mice. Individual scores of erythema, scale formation, and thickness were also significantly reduced upon treatment with rottlerin compared with the vehicle group (Fig 6D). The degree of splenomegaly is another characteristic of mice with IMQ-induced psoriasis-like inflammation. The mean spleen weight in the rottlerin-treated group of mice was significantly lower than that of vehicle group of mice (*P*<0.01; Fig 8E). In addition, H&E (hematoxylin-eosin) staining was used to assess the epidermal thickness and vascular density of the lesions. Histopathological changes in the skin of the vehicle group mice mainly included hyperkeratosis, acanthosis, and inflammatory infiltrates in the dermis, resembling human psoriasis. However, H&E staining of the dorsal skin sections from rottlerin-treated mice showed a distinct decrease in the incidence and severity of psoriasiform changes compared with the vehicle-treated mice. This decrease was characterized by diminished acanthosis and hyperkeratosis in the epidermis, as well as decreases in dermal vascular density. The histogram showed that rottlerin significantly decreased both the epidermal thickness and blood vessel area of the rottlerin-treated mice compared with vehicle-treated mice (both *P*<0.01; Fig 8F). Taken together, these results demonstrated that rottlerin improved the clinical and histological features of psoriasis-like skin inflammation in IPI mice.

### Rottlerin decreases inflammatory cell infiltration in IMQ-treated mice

**Fig 9:**
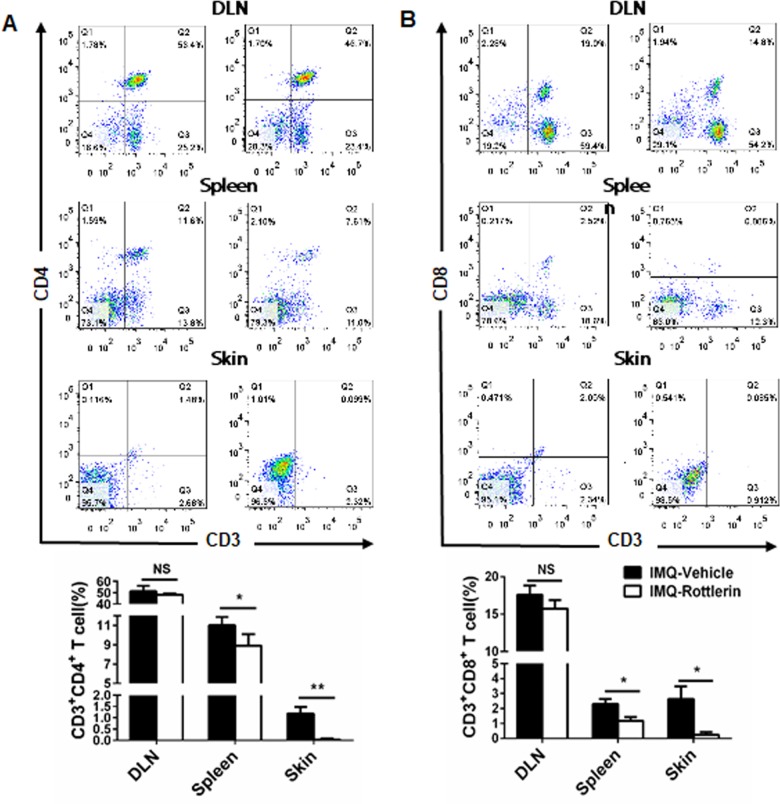
Rottlerin alters immunocytes compositions in IMQ-treated mice. An FACS analysis was applied to determine rottlerin activity following the oral administration of rottlerin or vehicle treatment in vivo. (A) Representative results of CD3+CD4+ T cell percentage in the DLN, spleen, and skin. The quantification of CD3+CD4+ T cell percentage is shown in the lower pannel. The data are represented as mean±SEM (n = 5 in each group). Similar results were seen in two other independent experiments. NS, *P*>0.05,*P<0.05,**P<0.01 vs. vehicle.(B) Representative results of the CD3+CD8+ T cell percentage in the DLN, spleen and skin. The quantification of CD3+CD8+ T cell percentage is shown in lower pannel. The data are represented as mean±SEM (n = 5 in each group). Similar results were seen in two other independent experiments. *P <0.05, ** *P*<0.01 vs. vehicle.

**Fig 10:**
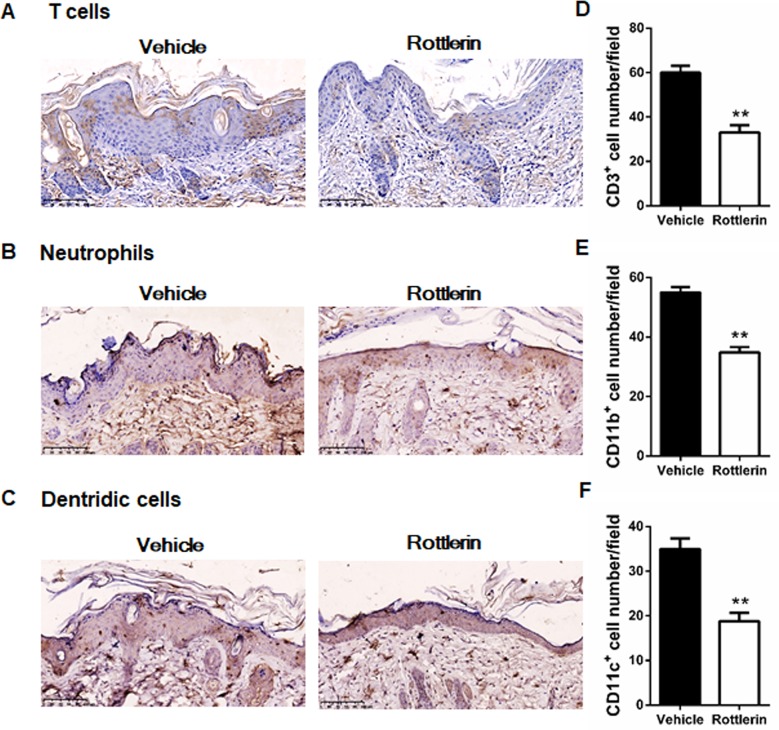
Rottlerin decreases the number of effector cells that mainly infiltrate the skin in IMQ-treated mice. Immunohistochemical detection of immune cell-related markers was performed on paraffin-embedded sections obtained from the back skin of IMQ-induced mice treated with vehicle or rottlerin. (A–C) Representatives IHC images of CD3 (A), CD11b (B), and CD11c (C) on the skin of the vehicle or rottlerin-treated mice. Scale bar = 100μm. (D–F) Quantification analysis of IHC staining for CD3(D), CD11b(E), and CD11c (F) on the skin of the vehicle and rottlerin treated mice. Two independent researchers counted the number of positive staining cells were per high-power field (HPF). The data are representative of three experiments (n = 5 mice per group).*P<0.05, ** *P*<0.01 vs. vehicle.

**Fig 11:**
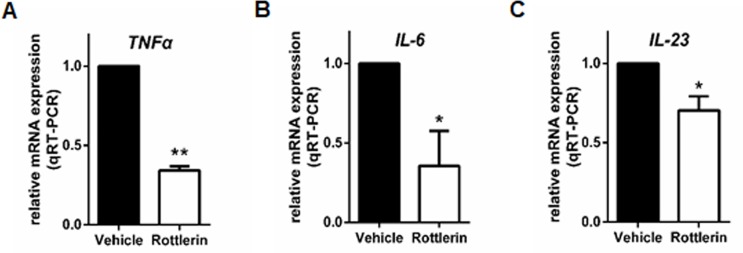
Rottlerin decreases the mRNA expression of inflammation genes in IMQ-treated mice. Relative mRNA expression levels in skin tissues from the vehicle and rottlerin treated mice were determined by qRT-PCR: (A) TNF-α; (B) IL-6; (C) IL-23. (n = 3, *P<0.05, **P<0.01 vs.vehicle).

On the basis that T cells play an important role in the pathogenesis of psoriasis, the infiltration of lymphocytes in the spleen, draining lymph nodes and skin were analyzed via flow cytometry. The percentages of CD3+CD4+T cells in the spleen and skin in the rottlerin-treated group of mice were significantly lower compared to that of the vehicle-treated group of mice (*P*<0.05 and *P*<0.01, respectively; Fig 9A). Similarly, the cellular compositions of CD3+CD8+T cells in the spleens and skin of the rottlerin-treated group of mice were significantly lower compared to those of the vehicle-treated group of mice (both *P*<0.01; Fig 9B). Although there was a decrease, there was no significant alteration in the cellular percentage of CD3+CD4+ and CD4+CD8+ T cells in the draining lymph nodes between the two groups of mice (*P*>0.05; Fig 9A and 9B). These results indicated that rottlerin attenuates the IPI of BALB/c mice, along with alterations in the compositions of immunocytes. Next, we profiled the immune cell infiltration in the rottlerin-treated group of mice and the vehicle-treated group of mice. We derived the skin tissues from the two groups by staining a number of immune cell markers, including CD3 (T cells), CD11b (neutrophils), and CD11c (dendritic cells). We observed that the CD3, CD11b, and CD11c immune-staining signals in the rottlerin-treated group were weaker compared with that of the vehicle-treated group (Fig 10A, 10B and 10C). The quantitative analysis of the number of positive cells stained by CD3, CD11b, and CD11c in the two groups also showed statistically differences (*P*<0.01; Fig 10D, 10E and 10F). In addition, to determine whether rottlerin decreases inflammation cytokines expression, we performed real-time PCR to compare TNF-α, IL-6 and IL-23 mRNA levels in the skin of both groups. The qRT-PCR results showed that, rottlerin reduced the mRNA level of TNF-α as compared to vehicle group mouse (*P*<0.01; Fig 11A). We also found that oral administration of rottlerin decreased IL-6 and IL-23 mRNA expression in psoriasis-like skin lesions (*P*<0.01; Fig 11B and 11C). Taken together, the administration of rottlerin reduced skin inflammation, which could potentially alleviate psoriasis-like skin lesions.

## Discussion

A growing amount of evidence suggests an anti-proliferation for rottlerin in the pathogenesis of cancer and immortalized cells, as well as vascular endothelial cells[19, 29, 30]. In this study, treatment with rottlerin induced a dose-dependent growth inhibition in NHEKs. Treatment with rottlerin also resulted in NFκB nucleus translocation inhibition. In agreement with previous findings[29], this result indicates that rottlerin interferes with the basal NFκB activation process, which also occurs in NHEKs. It is known that some drugs directly inhibit NFκB, thus regulating apoptosis. NFκB is a key regulator of immune responses and a crucial mediator of cell proliferation, migration, and apoptosis in various cell types[15, 31]. The inhibition of the NFκB activation process and DNA binding by rottlerin down-regulated the expression of cyclinD-1, which is an essential molecule for cells to advance from the G1 phase to the S phase of the cell cycle[17]. Moreover, as shown by immunofluorescence staining, treatment with rottlerin resulted in the increased translocation of p53 from the cytoplasm to the nucleus. The p53 tumor suppressor has been widely investigated and plays an important role in cellular processes, such as growth arrest, senescence, and apoptosis, in response to a broad array of cellular damage[32]. Taken together, the cell cycle arrest may be associated with the inactivation of NFκB and activation of p53 in the early stages.

Several previous studies has confirmed that rottlerin plays an essential role in the induction of autophagy and apoptotic cell death[14, 33, 34]. Autophagy is a catabolic process during which damaged organelles and proteins are engulfed and degraded to fulfill metabolic needs[35]. This process is activated in response to various kinds of stress and acts as a survival mechanism against aging and cellular senescence. However, autophagy can also lead to cell death in some circumstances. Rottlerin was found to cause typical autophagy characteristics in NHEKs at 24–48 h, as evident by the formation of autophagosomes, the redistribution of LC3, and induction of autophagy-related proteins, including Atg5, Atg12, and Beclin-1. 3-MA is a PI3K inhibitor that inhibits the fusion between autophagosomes and autolysosomes, which prevents the execution step of autophagy[36]. Our study demonstrates that 3-MA partially inhibited the rottlerin-induced conversion of LC3-I–LC3-II and the expression of autophagy-related proteins (Atg12 and Beclin-1) at 24 h, which suggests increased autophagic potential for rottlerin.

Apoptosis is an important tumor suppressor mechanism that is blocked in the majority of human cancers. Rottlerin was found to induce significant apoptosis in NHEKs at 48–72 h by inhibiting the expression of Bcl-2, the up-regulation of Bax, and the activation of cleaved Caspase-3. Meanwhile, rottlerin was found to cause typical autophagy characteristics in NHEKs at 24–48 h. Therefore, we conclude that the effect of rottlerin in inducing early-stage autophagy provided the potential mechanism of apoptosis induced by rottlerin in NHEKs.

In psoriasis, keratinocyte hyperproliferation goes along with aberrant terminal differentiation, resulting in parakeratosis and hyperkeratosis. To investigate the effect of rottlerin on keratinocyte proliferation and differentiation, we selected several markers that are relevant to abnormalities in psoriatic skin.miR-21 is reported upregulated in skin of psoriasis[37]. It is also widely studied as a biomarker in tumor-staging[38]. Yan et al. reported that transcription of miR-31 can be triggered by activated NFκB and then promotes the keratinocyte hyperproliferation in psoriasis[39]. Loricrin and involucrin are both important terminally differentiating protein of the epidermis[40]. Psoriatic lesions have previously been shown to exhibit up-regulation of involucrin and down-regulation of loricrin[9, 41]. We observed that rottlerin significantly reduced miRNA expression of miR-21 and miR-31 in a dose dependent manner. However, the mRNA levels of involucrin and loricrin were both significantly reduced after rottlerin treatment. Taken together, our results showed that rottlerin caused an alternation in the proliferation of keratinocytes, thus having a beneficial effect on psoriasis. But in another way, rottlerin treatment didn’t show a clear advantage in the regulation of differentiation.

We also investigated the effect of rottlerin on the third major hallmark of psoriasis: inflammation. As mentioned earlier, keratinocytes produce various cytokines during an inflammatory response, including TNF-α, IL-6, IL-17,IL-22, and IL-23[4, 5, 42]. Accordingly, we examined the anti-inflammatory action of rottlerin. We used TPA to stimulate cytokine production using keratinocytes because it is a known inducer of the keratinocyte inflammatory response[43]. Wallerstedt E et al. showed that rottlerin inhibits STAT3 signaling and blocks the transcriptional effect of IL-6[44]. Springael et al. showed that rottlerin blocked the differentiation of both Th1 and Th2 [45]. A variety of experiments have implicated NFκB as a key regulator of human cancer and inflammatory diseases[46, 47]. IKKα and IKKβ are both critical for cytokine-induced NFκB activation. Additionally, it has been reported that both IKKα and IKKβ can phosphorylate the p65 subunit to induce transactivation potential[48]. Furthermore, NFκB activation usually induces cytokines that regulate the immune response, and it is reported that inhibition of NFκB is a way to block inflammatory disorders[49]. Consistent with previous research results, based on qRT-PCR and ELISA data, our findings showed that rottlerin was a potent inhibitor of inflammatory cytokine expression through TPA-activated keratinocytes. Furthermore, rottlerin inhibited the expression of IKKα, IKKβ and NFκB p65 in keratinocytes, which indicates an anti-inflammatory effect by rottlerin treatment.

Angiogenesis acts an important role in the pathogenesis of psoriasis[50]. Angiogenesis is also required for cancer growth and invasion[51]. In this study, rottlerin reduced the HUVECs ability to form capillary-like structures on the matrigel, thus directly demonstrating anti-angiogenic properties.

In the healthy skin, there is a balance exists between pro-oxidants and anti-oxidants [52]. Without this balance, skin diseases, including psoriasis, become possible. Moreover, it has been found that anti-oxidants can provide innovative approaches to the management of psoriasis [53]. Previous studies have suggested that rottlerin exerts an anti-oxidative effect by decreasing ROS in MCF-7 cells and in HT-29 cells[18]. Our study indicated that rottlerin inhibited cell senescence and ROS in NHEKs, which suggested an anti-oxidation effect.

Utilizing the IPI model of psoriasis in vivo, we found that rottlerin attenuates IMQ-induced psoriasis after oral administration. Splenomegaly is considered as a characteristic of mice with IMQ-induced psoriasis-like inflammation [54]. We suggested that rottlerin may target the spleen through immune cell composition by inhibiting cellular proliferation. However, what exactly happens to the spleens was not clear. Rottlerin effectively limited the splenomegaly induced by IMQ and altered the percentages of CD3+CD4+ T cells and CD3+CD8+ T cells in the spleen and skin, while ameliorating of imiquimod-induced psoriasiform inflammation. Similarly, Springael et al. also observed the dose-dependent inhibition of CD4+ and CD8+ T cell proliferation in response to the stimulation of anti-CD3/anti-CD28 antibodies stimulation in the presence of rottlerin[45]. Sarkar K et al. demonstrated that antigen-specific proliferation stimulated by monocytes can be inhibited by rottlerin [55]. We also demonstrated that rottlerin attenuates IMQ-induced psoriasis-like inflammation by reduces infiltrations of CD3+T cells, CD11b+ neutrophils, and CD11c+ dendritic cells in the skin of IPI mouse model. However, this study lacks evidence of different concentrations of rottlerin in vivo, and the exact nature of rottlerin with other Th17/Th1 cytokines involved in immune responses is not clear. We conclude that the profound reduction of inflammation psoriatic animals treated with rottlerin is likely due to its combined effect on inflammation, keratinocytes proliferation and the proliferation of vascular endothelial cells.

In fact, there are conflicting results regarding inflammatory changes involving the inhibition of NFκB in mouse and human keratinocytes. Ulvmar et al. have shown that inhibiting NFκB activity in murine basal keratinocytes leads to inflammatory reaction and hyperproliferation[56]. However, there is also evidence that NFκB inhibition can be helpful in anti-inflammatory and antipsoriatic activities[43, 57].

Furthermore, rottlerin has been used for a long time in India as an anthelmintic agent, which suggests excellent drug safety[58]. Lu et al. have also carried out pharmacokinetics and tissue bioavailability research to study the efficacy and toxicity of rottlerin[26]. Used as an anti-tumor and renoprotection agent, rottlerin is administered orally in a single dose of 10–120 mg/kg in mouse models with cancer[26, 27]. Consistent with the preliminary results, our data also showed that rottlerin was well tolerated in animal studies with no significant changes in bodyweight, thus indicating that it is a relatively safe reagent. And, as noted, since there are no sufficient psoriatic keratinocytes available, one defect of the in vitro study is the use of normal keratinocytes, which means our results can only be regarded as a proof of concept regarding the effects of rottlerin in keratinocytes. In the future, researches are required to confirm this on psoriatic keratinocytes.

The present study clarified the effects of rottlerin in human primary keratinocytes with in vitro and in vivo IPI-induced psoriasis. In spite of rottlerin failed in differentiation restoring, it had been shown multiple potential effects on proliferation, inflammation and angiogenesis. We confirmed that rottlerin induced proliferation inhibition and apoptosis of keratinocytes. Moreover, rottlerin decreased the production of inflammatory cytokines by keratinocytes. Finally, rottlerin exhibited antioxidant and anti-angiogenesis effects. Our findings in the IPI mouse model in vivo suggested that rottlerin could lead to the amelioration of chronic T cell-dependent skin inflammation, reduction of skin thickening and angiogenesis. Although the detailed molecular mechanisms underlying the multiple anti-psoriasis effects of rottlerin remains largely unknown, our findings and data from the literatures suggest a potential use for rottlerin in the treatment and control of psoriasis.

## Supporting information

S1 Table Primers for differentiation markers and inflammation markers. (DOCX)
