# Supplementary material for: Rottlerin as a therapeutic approach in psoriasis: Evidence from in vitro and in vivo studies
Source: PLoS One. 2017 Dec 22;12(12):e0190051. doi: 10.1371/journal.pone.0190051 (PMC5741235; doi:10.1371/journal.pone.0190051)
Supplement: S1 Table — (DOCX) [file pone.0190051.s001.docx]

Supplementary Table 1. Primers for differentiation markers and inflammation markers

| Accession Number | Gene | Sense Primer | Anti-sense Primer |
| --- | --- | --- | --- |
| [NM_](https://www.ncbi.nlm.nih.gov/entrez/viewer.fcgi?db=nucleotide&id=119395749) 000427 | Loricrin | AGAAGCCATTGAGCTCTCCG | ACTGGGGTTGGGAGGTAGTT |
| [NM_005547](https://www.ncbi.nlm.nih.gov/nuccore/NM_005547.3) | Involucrin | CTGCCCACAAAGGGAGAAGT | AGCGGACCCGAAATAAGTGG |
| NM_000594 | TNF-α | CTGGGCAGGTCTACTTTGGG | CTGGAGGCCCCAGTTTGAAT |
| [NM_](https://www.ncbi.nlm.nih.gov/nuccore/NM_002190.2)000600 | IL-6 | CTCAATATTAGAGTCTCAACCCCCA | GAGAAGGCAACTGGACCGAA |
| NM_016584 | IL-23 | ACTTGTTGGGTGGCGTTAGA | TCCCATCTCTGGTCCCCATT |
| NM_001101 | ACTB | GATGACCCAGATCATGTTTG | CGTACAGGGATAGCACAG |
| NM_001278601 | Tnf-α | GATCGGTCCCCAAAGGGATG | CCACTTGGTGGTTTGTGAGTG |
| NM_001314054 | Il-6 | GACAAAGCCAGAGTCCTTCAGA | TGTGACTCCAGCTTATCTCTTGG |
| NM_031252 | Il-23 | AATGCTATGGCTGTTGCCCT | CACTGGATACGGGGCACATT |
| NM_007393 | Actb | TGAGCTGCGTTTTACACCCT | GCCTTCACCGTTCCAGTTTT |
